# Investigating the implementation of a novel approach to alcohol screening and brief intervention in Mexico: a mixed-methods study using pseudo-patients

**DOI:** 10.3389/fpubh.2024.1416190

**Published:** 2024-10-23

**Authors:** Deborah A. Fisher, Joel W. Grube, Liz Lilliott-González, Marissa Elias, Robert Sturm, Christopher L. Ringwalt, Ted R. Miller, Elena Cardenas Vargas, Tom Achoki, Angela Rizzo

**Affiliations:** ^1^PIRE Programs NF, Pacific Institute for Research and Evaluation, Beltsville, MD, United States; ^2^PIRE Programs NF, Pacific Institute for Research and Evaluation, Berkeley, CA, United States; ^3^PIRE Programs NF, Pacific Institute for Research and Evaluation-Southwest, Albuquerque, NM, United States; ^4^PIRE Programs NF, Pacific Institute for Research and Evaluation, Chapel Hill, NC, United States; ^5^Curtin School of Public Health, Australia University, Perth, WA, Australia; ^6^AB InBev Foundation, New York, NY, United States; ^7^Africa Institute for Health Policy, Nairobi, Kenya

**Keywords:** alcohol, screening and brief intervention, implementation, harmful drinking, pseudo-patients

## Abstract

**Introduction:**

Low- and middle-income countries bear disproportionate burdens from excessive alcohol consumption, yet have fewer resources to identify and intervene with risky drinkers. Low-cost screening and brief intervention (SBI) models offer a tool for addressing this health problem and reducing disparities.

**Methods:**

In this mixed-methods study, trained pseudo-patients visited health clinics in Zacatecas, Mexico, where a novel SBI model was used with trained nonmedical health educators (HEs) conducting SBI in waiting areas. Pseudo-patients, who provided responses to the AUDIT-C screening items designed to trigger a brief intervention (BI), waited for HEs to engage them in an SBI encounter. Data on HEs’ behaviors, SBI components provided, and contextual characteristics were coded from audio recordings of the encounters using an SBI checklist and from pseudo-patient interviews.

**Results:**

Quantitative analyses examined the consistency in pseudo-patients’ targeted AUDIT-C scores and those documented by HEs as well as the frequency of delivery of SBI components. Across 71 interactions, kappas between HEs’ scores and the targeted AUDIT-C scores ranged from 0.33 to 0.45 across AUDIT-C items; it was 0.16 for the total AUDIT-C. In 41% of interactions, the HEs recorded total AUDIT-C scores that accurately reflected the targeted scores, 45% were below, and 14% exceeded them. Analyses of checklist items and transcripts showed that HEs demonstrated desired interpersonal skills (attentive, empathetic, professional) and provided general information regarding risks and recommendations about reducing consumption. In contrast, personalized BI components (exploring pseudo-patients’ personal challenges and concerns about reducing drinking; making a plan) occurred much less frequently. Pseudo-patient interviews revealed contextual factors (noise, lack of privacy) that may have negatively affected SBI interactions.

**Discussion:**

Using trained nonmedical persons to administer SBI holds promise to increase its reach. However, ongoing training and monitoring, prioritizing comprehensive BIs, eliminating contextual barriers, and electronic delivery of screening may help ensure high quality delivery.

## 1 Introduction

Excessive alcohol consumption constitutes a global health problem, leading to 1.8 million deaths in 2020 (1). Since the 1980s, the World Health Organization (WHO) has promoted screening and brief intervention (SBI) as a strategy to address problem drinking, as have the National Institute on Alcohol Abuse and Alcoholism (NIAAA), the Centers for Disease Control and Prevention, and the Substance Abuse and Mental Health Services Administration (SAMHSA) in the United States (2–5). SBI is typically conducted in primary care settings where a health care professional provides a screening to identify patients and deliver a brief intervention (advice or counseling) to those who screen positive for hazardous drinking that places them at risk for harm (6). Those with more serious alcohol use disorders (AUDs) are referred to treatment services.

Best practices for SBI implementation have been developed by NIAAA to guide the brief intervention (BI) process. To help increase patients’ receptivity to advice and motivation for changing their drinking, the use of motivational interviewing (MI) principles is central. These principles include expressing empathy, exploring patients’ own reasons for reducing alcohol use, increasing their awareness of drinking consequences, and addressing resistance by affirming patient autonomy and self-efficacy (7, 8). MI techniques are used to engage collaboratively with patients and guide them through processes of identifying specific change goals, skills, and strategies to move toward these goals, and developing a personally tailored plan for reducing consumption (3, 9, 10).

Evidence regarding the efficacy of BI has produced generally a mix of significant but small effects and null effects (11–14). In a recent systematic review and meta-analysis, alcohol-targeted BIs delivered in general medical settings produced small reductions in alcohol use equivalent to about one drinking day per month; however, BIs yielded no effects on negative consequences (15). In contrast, results were inconclusive for alcohol-targeted BIs delivered in emergency departments/trauma centers.

In addition to modest effects, several systematic reviews have demonstrated decreasing effects over time for alcohol BIs in terms of both length of time since intervention and date of study publication. Results from a meta-analysis of randomized controlled trials conducted in low- and middle-income countries (LMICs) found that the intervention group had significantly lower scores on standard screening instruments at 3 months, but revealed no differences at 6- and 12-months post-intervention, suggesting attenuated effects over time (16). In a comparison of two Cochrane reviews, estimated effect sizes for reductions in alcohol consumption at 12 months declined from 41grams/week in the 2007 review to 20 grams/week in the 2018 review (17).

McCambridge (18) suggests that observed small/null effects and declining effect sizes result from a combination of theoretical weaknesses and empirical limitations. In particular, the mixed effects of SBI may reflect translational issues such as briefer interventions being implemented in practice compared to those conducted in the original efficacy studies (19). Despite potential issues with implementation, the delivery of BIs has received little research attention compared to studies of their efficacy and effectiveness.

Some studies have examined broad contextual moderators of BI effectiveness (e.g., length of time spent administering, use of boosters, delivery mode), but characteristics of SBI encounters at a more granular level have yet to be examined. It is unknown how SBI as conceptually envisioned compares to what is delivered. Documenting the real-life implementation of SBI can help clarify possible constraints on effectiveness and inform how necessary adaptations can best be implemented.

Understanding implementation is especially important as modes of delivery change. Research on health care provider barriers to conducting SBI (e.g., lack of training, concerns about their capabilities to deliver SBI, time constraints) have led to efforts to extend SBI to other health and non-health settings (20–22). In addition to addressing challenges faced by primary health care providers, efforts to use non-specialist health professionals and lay counselors are of particular interest in LMICs, where there is often an inadequate health care infrastructure and a pressing need for cost-effective measures to reduce alcohol-related harms (23, 24).

This study’s purpose is to examine the delivery of SBI in real-life settings to identify issues related to its implementation by trained nonmedical individuals in public settings in an LMIC. We used the SCALA framework underlying the SBI program in conjunction with widely endorsed guidelines on best practices for implementing SBI through motivational interviewing (7–10) to frame our study of implementation and assessments of the delivery of SBIs. To our knowledge, this study is the first to use pseudo-patients to collect detailed data on the content and contextual factors characterizing individual SBI encounters conducted by trained nonmedical persons.

## 2 Methods

### 2.1 Program description

The Escalemos project in the city of Zacatecas, Mexico, used trained nonmedical staff known as health educators (HEs), who had equivalent of a master’s degree in psychology and prior experience with substance abuse screening of youth in schools, to conduct SBIs with members of the public in the waiting areas of 10 local health care facilities (25). The project had been ongoing for about one year at the time our study was planned and conducted. Thus, the research team was not involved in the hiring or training of the HEs.

The Escalemos project is based on the SCALA framework, which has been used previously in Latin America and provides a step-by-step approach to implement alcohol SBI through primary health care at the local level^1^. Escalemos adopted this framework and adjusted it for implementation by non-medically trained persons. The project also employs the FRAMES approach, which comprises six components to guide patients toward self-awareness and build self-confidence to change (26). These components involve providing personalized Feedback to increase self-awareness of one’s AUD and its severity; encouraging patients to take Responsibility for their substance use choices as a means toward empowering patients to change; seeking permission to offer directive or educational Advice (e.g., suggestions that promote positive change); presenting choices through a Menu of options to promote engagement in the change process; demonstrating Empathy and understanding; and assisting with enhancing Self-efficacy by reviewing successes, personal strengths, and confidence-building to make positive changes (10).

HEs, wearing white lab coats emblazoned with the program’s name (“let us climb/rise” in English), approached individuals in waiting areas of health facilities. They introduced themselves and the project, and then invited the individuals to respond to the AUDIT (27), a standard screening tool that comprises questions about alcohol use. HEs recorded these responses on a tablet, which was connected in real time to a project dashboard used for managing the program.

Based on cross-cultural research conducted in Latin American countries, the Escalemos project adopted the scoring criteria of 1–7 on the AUDIT-C for providing patient education alone, and scores of 8 to 19 on the full AUDIT to trigger a BI (28). For screened individuals whose 3-item AUDIT-C scores fell between 1 and 7, the protocol called for the HE to provide feedback that acknowledged that the individual’s current drinking was considered low risk. However, they noted that because no level of consumption is completely safe it would be good to track consumption over time to keep it moderate. After this verbal feedback, the HE was instructed to give the individual an alcohol information handout (MAT10) that provided information on potential risk in comparison to the Mexican general population, standard drink sizes, moderate consumption standards by age and gender, potential harms of alcohol use especially pertinent to certain health conditions, and information concerning substance use treatment services. Non-drinkers were to be given positive feedback on their alcohol-free status and the same alcohol information handout. Individuals whose AUDIT-C scores were 8 or above were to be administered the additional seven AUDIT items. When scores on the full AUDIT fell between 8 and 19, the HE was to provide the individual with a BI that included feedback on their risky drinking, potential mental and physical health harms (especially in relation to any health conditions reported), advice about and goal setting for reducing use, and affirmation of their self-efficacy to reduce use. They were also instructed to give the individual an alcohol handout (MAT11) that included all the information specified in the MAT10 handout as well as a warning that the patient had scored as a risky drinker and a description of the benefits of reducing consumption, guidance on how to reduce harmful consumption, and a space to document a personal goal to reduce drinking.

Training for HEs conducted by the Escalemos project consisted of 20 h across five sessions in which different topics were presented including general concepts, administration of the AUDIT, delivery of brief advice, flow charts describing intervention pathways, and equipment use. These topics were presented and practiced in role plays and discussed in review sessions. Subsequently, the HEs went into the field to gain experience conducting SBIs with real patients. These interactions were observed by their supervisor and intensively evaluated on a daily basis with feedback provided after each interaction; after all errors had been corrected, the evaluations were conducted weekly as implementation progressed. This intensive review process lasted approximately three months.

Ongoing training and monitoring efforts included continuing intermittent evaluation by means of unannounced site visits by the supervisor to observe performance and provide feedback. Additionally, performance was monitored through reviews of data in the project’s digital dashboard (e.g., time spent interacting with an individual, responses to the screening items, and the brief interventions administered). On a monthly basis, HEs and their supervisor met to address problems and, as necessary, adapt aspects of the program in response to exigencies in the field.

### 2.2 Data collection

#### 2.2.1 Procedures

##### 2.2.1.1 Pseudo-patients

In collaboration with T.G. Consultoría (TGC), the local contractor that managed the SBI intervention, we recruited and trained six males and four females aged 22 to 49 (mean = 33.4 years) to serve as pseudo-patients. The role of pseudo-patients was to visit the waiting rooms of health facilities where SBI was being conducted and wait to be approached and screened by an HE to gather interaction-level data on the content and context of SBIs. Our use of pseudo-patients was necessary to standardize each SBI encounter to trigger a BI, and record each interaction without the knowledge of the HEs so the research team could later code both the screening and the brief intervention. Although the use of pseudo-patients in research has both advantages and risks (29), we saw no feasible alternatives to obtain the data required to address our research question. In this case, the deception involved was mitigated by including a clause in the HEs’ employment contracts clearly stating that monitoring and recording of SBI interactions without warning may occur.

##### 2.2.1.2 Pseudo-patient protocols

For their SBI encounters, each pseudo-patient, in consultation with research staff, developed a discrete culturally credible and personalized alcohol use persona or backstory (e.g., a twenty-something-year-old college student who drinks heavily only on weekends with her boyfriend and friends). These personas, and the answers each pseudo-patient provided to the alcohol consumption screening items, were designed to result in a 3-item AUDIT-C score between 8 and 10 and thus trigger a BI. To avoid referral to treatment, responses to the full AUDIT were designed so that total AUDIT scores did not exceed 15.

The selection criteria for pseudo-patients were that they must be residents of the state of Zacatecas, able to develop a culturally believable persona as a risky drinker, and convincingly maintain it throughout all their interactions with the HEs. This required that they follow the script for the screening that was developed individually with each pseudo-patient and the PIRE trainers and respond to the AUDIT items so as to generate scores within the targeted ranges and thus elicit a brief intervention.

Training was conducted over the course of four days. During the first two, trainers provided an overview of the research and the data collection task. They also worked with pseudo-patients to develop their alcohol use profiles, their target responses to the AUDIT items to elicit a brief intervention, and role playing of SBI interactions. During the next day and a half, pseudo-patients conducted preliminary visits to clinics in pairs. One person conducted a practice SBI interaction with an HE, who was unaware that the person they were engaging with was a pseudo-patient, while the other observed; in the next clinic visit, their roles as pseudo-patient and observer were reversed. Audio recordings from the practice encounters were reviewed by trainers and feedback was provided. On the second half of the fourth day, a group meeting was held for debriefing, making final adjustments to scripts, and wrap up.

Ongoing monitoring and feedback continued throughout the data collection process. A member of the training team maintained close communication with the field manager in Zacatecas, with whom pseudo-patients shared reflections on their field work as they turned in their devices after each SBI encounter so she could download the recordings. At that time, the sound quality of the audio recordings was checked. At two and three weeks into the field work, online meetings for trouble shooting and monitoring were held with the pseudo-patients, the PIRE training team, and the coordinator and the field manager in Zacatecas. The purpose of the meetings was to discuss any issues that had arisen and provide suggestions and feedback for improvements. Examples of these suggestions for pseudo-patients included adjustments to their use of the recording devices to obtain higher quality recordings, and behaviors that might increase their approachability such as locating themselves in more convenient spaces and wearing clothing that better reflected that of the population served by the clinics.

Pseudo-patients visited the health facilities at a time when an assigned HE was present. Each pseudo-patient was scheduled for SBI encounters to ensure they would be screened only once by each HE. Pseudo-patients entered the waiting areas and mingled with other patients and family members. Before entering the waiting area, pseudo-patients turned on two recording devices—a cell phone and a portable digital recorder—to capture their interaction with the HE. Although the cell phone was visible and in use as the pseudo-patient waited to be approached by an HE, the digital recorder was concealed. Both devices were meant to be used in such a way that HEs were unaware that their encounter with the pseudo-patient was being recorded.

After their encounter with the HE, pseudo-patients left the health facility and completed an SBI checklist designed to capture data on what happened during the interaction. Then the pseudo-patients returned to the TGC office so the coordinator could download the recordings and remove them from the devices.

One of the nine HEs dropped out prior to the start of the data collection, and another dropped out during the study. Eight of the HEs (three males, five females ranging in age from 27 to 62 years) are represented in the 74 SBI interactions that were conducted. Pseudo-patients conducted no more than two visits each week over the course of data collection, which occurred between mid-January and early April 2023.

All research procedures were reviewed and approved by PIRE’s Institutional review board (IRB) (FWA00003078). Research ethics review in Mexico is not required for studies involving social and behavioral research and thus the protocol was not submitted for further human subjects review. However, PIRE’s IRB requires a description of the local research context and discussion of how the research addresses the legal and cultural context and the linguistic needs of the population to be involved in the data collection. Additionally, in their employment contracts, HEs agreed to participate in project evaluation without prior notice, which they were informed could involve recording and monitoring of SBI interactions by their supervisor, the HE coordinator, or external personnel authorized by him. Given these agreements, HEs’ interactions with pseudo-patients were undertaken in the course of their normal professional responsibilities and no personal information was sought from them in the course of the SBI encounter. Due to the study’s nature, there was no involvement of patients or the public in its planning or conduct.

##### 2.2.1.3 Pseudo-patient debriefing interviews

Starting two weeks after the completion of the fieldwork, we conducted voluntary and confidential video interviews with each pseudo-patient. Two bi-lingual staff jointly co-facilitated interviews that lasted about 90 min. While one interviewer led the discussion, the other took summary notes in real time, which were reviewed and supplemented by the lead interviewer following the interview. Interviewing in teams ensured that all key topics would be addressed and facilitated a team-based analytical approach.

#### 2.2.2 Measures

##### 2.2.2.1 AUDIT data

HEs entered the scores they assigned to each AUDIT item on a tablet for all patients they engaged with, including the pseudo-patients. These scores, along with the HEs’ unique ID, location, date, and time, were uploaded into TGC’s dashboard. These data were downloaded and matched with each pseudo-patient/HE encounter. In addition, a difference score was calculated by subtracting the pseudo-patient’s targeted AUDIT-C score from the sum of the scores on the individual AUDIT-C items recorded by the HE.

##### 2.2.2.2 SBI checklist

Based largely on the NIAAA guidelines (3), we developed a checklist to document what occurred during each SBI encounter (see Table 1). The SBI checklist was designed to capture both specific components delivered as part of the BI (e.g., discussion of benefits of reducing alcohol consumption, personalized solutions to challenges of reducing drinking, development of a goal and a personally tailored plan for reducing alcohol use) and the HE’s style of interacting with the pseudo-patient to deliver the BI (e.g., empathetic and non-judgmental, attentive to the pseudo-patient). All items were coded Yes/No.

**Table 1:** Pseudo-patient checklist (response options yes/no to items 1–19).

1	Was the interaction with the HE private enough where others could not hear your responses?
2	Did the HE ask about your specific health conditions?
	Did the HE administer the following 3 questions from the AUDIT-C?
*3*	*How often do you have a drink containing alcohol?*
*4*	*How many drinks containing alcohol do you have on a typical day when you are drinking?*
*5*	*How often do you have six or more drinks on one occasion?*
6	Did the HE provide the MAT11 handout?
7	Did the HE talk to you about health risks (mental or physical) associated with alcohol use?
8	Did the HE talk to you about any benefits of reducing alcohol use (cutting down)?
9	Did the HE provide examples of the benefits of reducing your alcohol use for your specific health conditions?
10	Given concerns about cutting back or quitting, did the HE help you identify solutions?
11	Did the HE ask if you were ready to change?
12	Did the HE develop a goal with you about reducing your alcohol consumption?
13	Did the HE help you develop a plan to achieve your goals for reducing your alcohol use?
14	Did the HE ask if you understood the information they were providing?
15	Did the HE appear to be listening carefully to what you were saying?
16	Was the HE empathetic and non-judgmental?
17	Did the HE stay on topic (e.g., did not wander or bring up irrelevant topics)?
18	Did the HE summarize what you discussed?
19	Did the HE review next steps with you?
20	Who initiated the SBI interaction? 1 = The HE 2 = Pseudo-patient
PP to document by adding at the end of audio recording	Did anything notable or unusual happen during your interaction with the HE that disrupted the interview and provides important context such as a loud argument, equipment problem (HE’s iPad, one of your audio recording devices), etc.? If YES, please provide a verbal description at the end of the recording for the encounter and notify the field manager immediately.

An overall SBI score was calculated by taking a count of the BI component that each HE completed for each encounter with a pseudo-patient. The extent that HEs completed the general BI components was quantified by taking a count of 8 items (asked about health conditions, provided the AUDIT score, explained the AUDIT result, provided the MAT11, discussed general physical health risks, discussed general mental health risks, discussed general benefits of reducing drinking, and recommended reducing drinking). Completion of a more personalized BI using MI strategies was assessed by taking a count of the remaining 10 items (asked about personal reasons for reducing drinking, discussed personal benefits of reducing drinking, asked about the pseudo-patient’s concerns about reducing, offered solutions to those concerns, helped develop goals, helped develop a plan to meet those goals, asked if the pseudo-patient was ready to change, asked if the information was understood, summarized the information, and reviewed next steps).

Following the transfer of the audio recordings of the SBI encounters to the research team, one of our three Spanish-and-English-fluent staff used NVIVO Transcribe to identify and correct auto transcription errors. The constructs in the SBI checklist were then coded by the three bi-lingual research team members, who used both the audio recordings and transcripts. The primary coder coded all the interactions. The two other team members then reviewed these codes while listening to the recordings and reading transcriptions, focusing on areas flagged by the first coder as difficult to code. Any disagreements were resolved by collective review and consensus.

The coders’ ratings were used for the objective items on the checklist (e.g., correct administration of the AUDIT, inclusion of key components of the BI). For subjective items relating to the HEs’ style of interaction that depended on visual cues (e.g., body language, eye contact), which would not be available to the coders, we used the pseudo-patients’ responses.

##### 2.2.2.3 Pseudo-patient debriefing interviews

A semi-structured interview protocol organized topically with follow-up questions and probes was developed to explore pseudo-patients’ experiences of the SBI encounters and to elicit their assessments of the benefits and challenges of the model (i.e., implementation in public spaces) used to deliver it (Appendix A). The semi-structured format engaged participants in a guided yet free-flowing discussion in which they were invited to share relevant thoughts and perceptions.

#### 2.2.3 Data analyses

##### 2.2.3.1 Quantitative analyses

Quantitative analyses consisted of crosstabs to compare the HEs’ recorded total and individual item scores on the AUDIT-C obtained from the dashboard with the pseudo-patients’ targeted scores. Weighted kappas were used to assess the extent of agreement between these scores. Frequency analyses were used to investigate whether the HEs addressed each of the items on the SBI checklist. We used generalized linear models to explore differences in targeted and recorded AUDIT-C scores and differences among the HEs in the number of SBI components they successfully completed. We also explored differences by HE and pseudo-patient gender. Poisson models were used for count variables (SBI scores) and linear models were used for the AUDIT-C difference scores. We conducted all quantitative analyses using SPSS version 29.

##### 2.2.3.2 Qualitative analyses

Rapid Qualitative Data Analysis (30), an approach often employed for the efficient identification and synthesis of key findings across datasets, was used. A matrix containing pseudo-patient ID numbers and the core interview constructs was developed and stored in a shared online site for interviewers to record aggregate interview notes. This matrix allowed the team to quickly review pseudo-patients’ collective responses, get a sense of variation and gaps in information they provided, and highlight insights about emerging themes and patterns (31).

## 3 Results

### 3.1 Quantitative findings

Of 74 interactions completed between HEs and pseudo-patients, AUDIT-C data from the dashboard were available for 71. SBI checklist data were successfully coded for 61 (82%) of the interactions. Coding was not possible for 13 interactions because background noise, participants’ use of face masks, or equipment issues made the recordings inaudible.

#### 3.1.1 AUDIT-C

##### 3.1.1.1 AUDIT-C total scores

Table 2 compares the total targeted AUDIT-C scores the pseudo-patients were trained to generate and the scores recorded by the HEs. Overall, agreement between the total scores based on the pseudo-patients’ scripts and those recorded by the HEs was very poor (*κ* = 0.16). Whereas the pseudo-patients were trained to give responses to the AUDIT-C items designed to result in scores between 8 and 10, the HEs recorded scores ranging from 5 to 11. Overall, in 41% of the interactions, the recorded total score accurately reflected the targeted score. For 45% of the interactions, the recorded total scores were below the targeted score, and for 14% they exceeded it. For 30% of the interactions, the pseudo-patients were scored below 8 and thus did not meet the threshold for a brief intervention.

**Table 2:** Comparison of pseudo-patient targeted AUDIT-C scores and scores recorded by HEs.

Recorded score	Pseudo-patient targeted score		
	8	9	10
5	1 (2.9%)	—	—
6	4 (11.4%)	2 (7.7%)	2 (20%)
7	7 (20.0%)	4 (15.4%)	1 (10%)
8	18 (51.4%)	6 (23.1%)	3 (30%)
9	2 (5.7%)	9 (34.6%)	2 (20%)
10	2 (5.7%)	4 (15.4%)	2 (10%)
11	1 (2.9%)	1 (3.8%)	—
Weighted κ = 0.16			

Table entries are column counts with percentages in parenthesis.

##### 3.1.1.2 AUDIT-C difference scores across HEs

Analysis of the AUDIT-C difference scores showed a significant effect by HE, Wald χ^2^(8) = 47.65, *p* < 0.001. On average, all HEs underestimated the AUDIT-C score (mean = −0.59). One HE underestimated the scores to a greater extent (mean = −1.67) than the other HEs, Wald χ^2^(8) = 37.50, *p* < 0.001. The other HEs did not differ significantly from one another.

##### 3.1.1.3 AUDIT-C difference scores by pseudo-patient gender

Pseudo-patient gender was not significantly associated with the AUDIT-C difference scores, Wald χ^2^(1) = 0.26, *p* = 0.61. The means for females and males were − 0.69 and − 0.52, respectively.

##### 3.1.1.4 AUDIT-C item scores

Based on the checklist data, although the AUDIT-C drinking frequency and typical quantity items were asked correctly in a great majority of interactions (100 and 98%, respectively), the heavy drinking item (frequency of 6+ drinks per occasion) was asked correctly only 46% of the time. Table 3 shows a comparison of the pseudo-patients’ targeted scores on each of the AUDIT-C items and the scores that were recorded by the HEs. Agreement between assigned scores and recorded scores was modest, with weighted kappas ranging from 0.33 to 0.45. For drinking frequency (AUDIT 1), 59% of the interactions were coded correctly, 32% were recorded at a lower category than targeted, and 9% were coded in a higher category. For typical quantity consumed (AUDIT 2), 55% of the interactions, overall, were coded correctly, 34% were coded in a lower category than targeted, and 11% were coded in a higher category. Finally, for the heavy drinking item (AUDIT 3; frequency of consuming 6+ drinks) 76% of interactions, overall, were recorded accurately, 10% were coded in a lower category than targeted, and 14% were coded in a higher category.

**Table 3:** Comparison of pseudo-patient targeted AUDIT-C item scores and scores recorded by HEs.

Score recorded by HE	Pseudo-patient targeted score		
AUDIT 1: Drinking frequency	2–4 times a month	2–3 times a week	4+ times a week
Monthly or less	1 (7.7%)	1 (2.3%)	—
2–4 times a month	12 (92.3%)	14 (31.8%)	—
2–3 times a week	—	23 (52.8%)	7 (50.0%)
4+ times a week	—	6 (13.6%)	7 (50.0%)
Weighted κ = 0.45			
AUDIT 2: Typical drinks per occasion	5–6 Drinks	7–9 Drinks	10+ Drinks
1–2 drinks	—	3 (10.0%)	—
3–4 drinks	1 (3.3%)	2 (6.7%)	—
5–6 drinks	22 (73.3%)	14 (46.7%)	1 (9.1%)
7–9 drinks	7 (23.3%)	10 (33.3%)	3 (27.2%)
10+ drinks	—	1 (3.3%)	7 (63.6%)
Weighted κ = 0.34			
AUDIT 3: Frequency 6+ Drinks	Monthly	Weekly	—
Less than monthly	—	2 (3.1%)	—
Monthly	6 (85.7%)	5 (7.8%)	—
Weekly	1 (14.3%)	48 (75.0%)	—
Daily or almost daily	—	9 (14.1%)	—
Weighted κ = 0.33			—

Table entries are column counts with percentages in parentheses.

#### 3.1.2 SBI checklist

##### 3.1.2.1 Style of SBI delivery

Characteristics of HEs’ style of delivering the SBIs were uniformly rated highly by pseudo-patients. In all or nearly all interactions, the HEs were perceived as being empathetic and nonjudgmental (100%), staying on topic (100%), and listening carefully (96%).

##### 3.1.2.2 SBI components delivered by HEs

**Figure 1:**
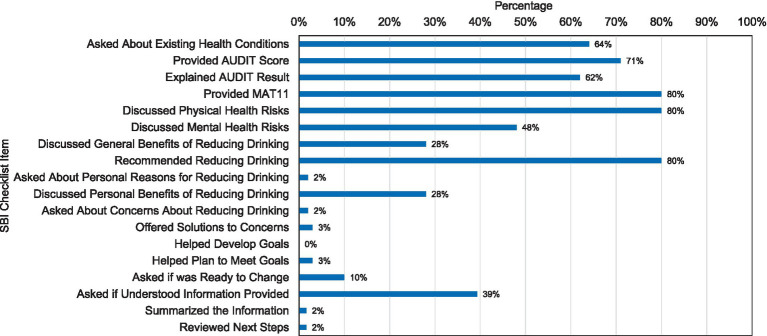
Percentage of times health educator addressed screening and brief intervention elements.

The percentage of interactions where the HEs adequately addressed each of the SBI components was much more variable as is displayed in Figure 1. The HEs addressed some SBI components most of the time, but not consistently. They provided the MAT11 to the pseudo-patient 80% of the time overall and 94% of the time when the HE scored the pseudo-patient 8 or higher on the AUDIT-C. In about 60 to 80% of interactions, they asked the pseudo-patient about general health conditions (64%), provided an AUDIT-C score (71%), explained the AUDIT-C result (62%), discussed physical risks of drinking (80%), and recommended reducing drinking (80%). None of these key components of SBI, however, were implemented in all interactions. Other components of SBI were implemented in less than half of the interactions. These included discussed mental health risks (48%), asked if the pseudo-patient understood the information provided (39%), discussed general benefits of reducing drinking (28%), discussed personal benefits of reducing drinking (28%), and asked if they were ready to change (10%). Other strategies occurred in two or fewer interactions, including helped the pseudo-patient make a plan to meet drinking goals (3%), offered solutions to the pseudo-patient’s concerns about reducing their drinking (3%), asked about the pseudo-patients’ reasons for reducing drinking (2%), asked about personal concerns about reducing drinking (2%), summarized the information discussed (2%), and reviewed next steps (2%). There were no instances in which the HEs helped the pseudo-patient develop goals to reduce drinking.

##### 3.1.2.3 Differences in SBI scores among HEs

Table 4 shows the means for the HEs on the three SBI scales. Considering the total score, on average, HEs scores ranged from 1.7 to 8.6 out of a possible score of 18 (mean = 6.03). There was a statistically significant difference among the HEs in the average total number of SBI components completed across encounters, Wald χ^2^(7) = 36.44, *p* < 0.001. Three of the HE’s scored significantly lower than the others. The other HEs did not differ significantly from one another. Considering the general SBI score, the means ranged from 1.3 to 6.8 out of a possible score of 8 (mean = 5.13). There was a statistically significant difference among HEs, Wald χ^2^(7) = 24.74, *p* < 0.001, with one HE lower on this measure than the other HEs. For the personalized BI measure, mean scores ranged from 0–1.9 out of a possible score of 10. On average, HEs completed less than one of these components across encounters (mean = 0.9). There again was a significant difference among the HEs, Wald χ^2^(7) = 12.89, *p* < 0.05, with one HE never completing any of the components on any occasion and two scoring significantly lower than other HEs.

**Table 4:** Health educator scores on the screening and brief intervention scales.

HE	Overall score		General SBI items		Personalized SBI items	
	Mean	*p*	Mean	*p*	Mean	*p*
1	7.0	0.26	5.7	0.43	1.3	0.37
2	7.6	0.45	6.4	0.77	1.2	0.25
3	5.2	< 0.01	5.0	0.13	0.2	< 0.01
4	7.0	0.25	5.6	0.37	1.4	0.43
5	4.6	< 0.01	4.6	0.08	0.0	--
6	1.7	< 0.001	1.3	< 0.001	0.43	< 0.05
7	5.7	0.12	4.7	0.22	1.0	0.32
8^a^	8.6	--	6.8	--	1.9	--

^aReference category. Main table entries are mean counts of the number of SBI components completed. Total = 18 items; General BI = 8 items; Personalized BI = 10 items.^

##### 3.1.2.4 Differences in SBI scores by HE gender

There was a small difference between male and female HEs on the overall scale, Wald χ^2^(1) = 4.27, *p* < 0.05, with females (mean = 6.5) tending to complete more of the components than males (mean = 5.1). There was also a small effect of HE gender for the general BI measure, Wald χ^2^(1) = 4.41, *p* < 0.05, with females (mean = 5.6) addressing more of the components, on average, than males (mean = 4.3). Females and males did not differ significantly on the personalized BI scale, Wald χ^2^(1) = 0.27, *p* = 0.61.

##### 3.1.2.5 Differences in SBI scores by pseudo-patient gender

There were no statistically significant differences between female and male pseudo-patients in terms of the components received on any of the SBI scales. For the overall scale, Wald χ^2^(1) = 0.53, *p* = 0.47, the mean for females was 5.7 and for males it was 6.2. For the general BI scale, Wald χ^2^(1) = 0.06, *p* = 0.81, the mean for females was 5.0 and for males 5.2. For the personalized BI scale, Wald χ^2^(1) = 1.72, *p* = 0.19, the means for females and males were 0.7 and 1.0, respectively.

### 3.2 Qualitative findings

Pseudo-patients were asked to reflect on their personal experiences with HEs as well as their insights based on their observations of interactions between HEs and clinic patients. Themes emerged regarding what worked well and where challenges were encountered in delivering SBI as implemented in the current Escalemos protocol.

#### 3.2.1 Theme 1

*Health educators were perceived as conducting themselves in a professional and respectful manner when administering SBI*. As one pseudo-patient explained, “They were very conscientious public servants, and they knew how to treat people, how to get their attention, how to explain [the results]…” (Pseudo-patient #6). Another echoed these sentiments saying, “They paid close attention to what I was saying in order to give me personalized advice” (Pseudo-patient #5). Several pseudo-patients commented that the medical white coat worn by the HEs contributed to their being perceived as a credible source of information.

#### 3.2.2 Theme 2

*Health educators delivered relevant information on alcohol and health in a way that pseudo-patients found easy to understand*. Most pseudo-patients agreed that the information provided was easily accessible. For example, one said, “They explained it in a way… anybody could understand this information” (Pseudo-patient #10). Others mentioned that the HEs’ guidance on how to reduce drinking was helpful and often new, interesting, and relevant to their own experiences. As one pseudo-patient explained, “As far as the information from the screening, there were a lot of things that I did not know -- one thing that stood out to me a lot was not consuming salty foods when you are drinking” (Pseudo-patient #5).

#### 3.2.3 Theme 3

*Pseudo-patients often found the intervention settings to be crowded with considerable background noise and a general lack of privacy that made the SBI difficult to conduct*. Several expressed concern that the contextual factors might reduce patients’ willingness to fully participate in the screening or to answer honestly. They mentioned the presence of family members, especially children, other patients, and ambient noise when the SBI was in an indoor and crowded location. These environmental factors were perceived to interact with and reinforce a general cultural norm inhibiting discussing one’s alcohol use and potential problems in public. When reflecting on how they saw others respond to the intervention, one pseudo-patient stated that “The majority responded in a polite way, but most of them said no, they do not consume… so as to avoid the longer interaction…” (Pseudo-patient #1). Another reflected, “I was able to see that people from the [rural/Indigenous] communities, well they are very timid, and so I saw that [when] the [HE] approached them to ask them questions … [the patients] avoided them” (Pseudo-patient #10).

#### 3.2.4 Theme 4

*Encounters often felt rushed, which may have limited the exchange of information*. This theme was reflected in statements when the pseudo-patients indicated that they did not get the full BI or that AUDIT screening items were skipped or paraphrased. Pseudo-patients presumed several factors led to rushed interactions, including HEs’ fatigue and desire to end their shift. For example, “I was the last one… I waited maybe two hours there, but they took five minutes with me. It was obvious to me that they wanted to leave. I was the last one to reach their goal [quota]” (Pseudo-patient #9). Others suggested, “Maybe they were in some sort of rush and were a little fed up with the day and they were very fast, they asked me the first two questions, gave me the MAT10 and then left” (Pseudo-patient #1) and “I think in two or three of the encounters where I felt that they were lacking… maybe they started off on the wrong foot, they were not really feeling up for it, or it was a hard work day” (Pseudo-patient #6). Another reason offered was the difficulty conversing due to mandatory face masks. “They had the face mask, and we were also required to have it and so maybe that hindered communication a little.” (Pseudo-patient #8).

## 4 Discussion

Although LMICs have a higher alcohol-attributable disease burden than well-resourced countries, specialized services for AUDs are limited or non-existent (16, 32). Given that much of the projected increase in alcohol consumption and harmful use of alcohol is expected to occur in LMICs, cost-effective strategies are needed to address these alcohol-related health disparities, including the potential to employ non-specialist health workers and lay counselors, to deliver interventions for a range of disorders, including AUDs (23). Our findings concerning the delivery of SBI by HEs in Zacatecas, Mexico, can help inform the planning and implementation of programs designed to bring AUD services to at-risk populations in LMICs.

Our quantitative and qualitative findings regarding the delivery of SBI by trained HEs in waiting areas of public health facilities suggest that several aspects of the intervention worked well. HEs were described as professional and respectful, delivering relevant information about alcohol use and health in a way that was easy to understand. In all or nearly all interactions, pseudo-patients reported that HEs stayed focused, were empathetic and nonjudgmental, listened attentively, discussed general physical health risks, and recommended reducing drinking. Additionally, in more than three-quarters of interactions overall, HEs provided the MAT11 alcohol information and resource handout to the pseudo-patient. They provided it in 94% of the encounters when they scored the pseudo-patient 8 or higher on the AUDIT-C, suggesting the failure to provide it was largely a matter of mis-scoring the AUDIT.

Our findings also revealed challenges in delivering some components of SBI. With respect to screening, in more than half of the cases, the total AUDIT-C scores recorded by HEs were not consistent with the total scores that pseudo-patients should have generated from the information they provided. Most often, the HEs’ recorded scores were lower than the pseudo-patients’ targeted scores. Although in some cases a difference of one or two points in the total score was inconsequential, for 30% of the interactions, the pseudo-patients were scored below the criterion of 8 and thus received neither the additional screening items nor the brief intervention. As with AUDIT-C total scores, there was considerable variability in individual AUDIT-C item scores, indicating incongruence between responses targeted by pseudo-patients and those recorded by HEs. There was also variability in the accuracy of administering the AUDIT-C questionnaire, insofar as the items were administered correctly in nearly all cases for frequency of drinking and typical quantity consumed, but in less than half the cases for the frequency of consuming six or more drinks. Additionally, in some cases, pseudo-patient’s response to an earlier AUDIT-C item may have led an HE to break protocol and not ask a later screening item. For example, in answering the second item on typical quantity consumed if a pseudo-patient said they usually had three drinks, the HE may have assumed they never had 6+ drinks and not administered the third item on the frequency of heavy episodic drinking (i.e., consuming 6 or more drinks on an occasion). If the administration and scoring of the AUDIT were automated, this possibility for error would be eliminated. Finally, HEs often did not read the response options to the AUDIT questions. In addition, rather than probing the conversational answers pseudo-patients were instructed to give in order to create a natural dialog, the HEs likely inferred the AUDIT response, in some cases introducing errors in the scoring.

The target AUDIT-C scores for pseudo-patients (8–10) were at the lower end of the scoring continuum that should have prompted a BI given the Escalemos protocols (8 or higher). Thus, mis-scoring by HEs resulted in nearly a third of interactions producing an AUDIT-C score below the threshold for a BI. We could have set the target scores higher, and it is possible that more interactions that were underscored by one or two points may have resulted in the HEs providing a BI. However, SBI is used with the general population, and high AUDIT-C scores are relatively infrequent. Additionally, given that the purpose of our study was to examine issues concerning implementation of this novel SBI program, the study design allowed us to identify potential problems and implications for both screening and the delivery of BIs. It was, in fact, the case that a substantial number of pseudo-patients who should have scored high enough to clearly need an intervention did not receive one.

The findings regarding discrepancies in the screening reinforce the importance of ongoing training to ensure that the assessments are conducted correctly. Although those administering the SBI may be tempted to reword items to be less formal or intimidating, provide clarification, establish rapport, or avoid asking a question whose answer seems obvious, such changes risk undermining the validity of the assessments and thus the delivery of the BI, which is predicated on the screening score. Overall, these findings indicate that if scoring is not automated, SBI training for HEs should emphasize the importance of administering all the required AUDIT items as written.

Related to training is the issue of implementation drift. Variation in program delivery over time is an issue often encountered, but it is especially relevant when an intervention is relatively complex and relies on individuals who may lack extensive experience with the protocol. Ongoing monitoring and providing feedback on a continual basis seems essential. Methods for achieving this could include unobtrusive observations of pseudo-patient interactions to capture the tone and content of the exchanges, and group meetings to discuss and resolve issues to minimize deviations from the intervention protocols. Group meetings also provide a collegial forum for those administering SBI to share challenges and, in collaboration with trainers, develop strategies for responding to them. These methods may also be critical to identifying unanticipated events occurring in the process of administering SBI so they can be addressed as promptly as possible.

Regarding brief interventions, although overall HEs demonstrated the desired interpersonal skills and succeeded in providing general risk information and recommending efforts to reduce risky consumption, other critical characteristics of MI were not present in most encounters. These were typically in areas that involved engaging pseudo-patients in a more in-depth discussion about changing their behavior, including discussing their potential reasons for reducing their drinking; their personal strengths, challenges, and concerns; setting a goal; and helping them make plans to meet their goal. Despite the potential benefits of using MI to personally tailor the components of BIs, by nature this process requires more time and effort than providing patients with general alcohol risk information and advice. The low frequency with which these MI components were delivered by HEs may suggest the need for more training or the need for an approach to SBI that places more emphasis on MI than does the FRAMES approach as implemented by the Escalemos program. Additionally, the clinic settings for the SBI program posed multiple challenges such as noise, interruptions, appointments that could start at any time, and a lack of privacy that made conversing difficult in some cases and hindered the SBI process. It is likely that a combination of these factors contributed to the low rates of implementation of personalized BI components. Efforts to ensure that BIs include the development of a personalized goal and strategies include (1) allotting adequate time to conduct BIs with all the components; (2) providing conducive settings (i.e., quiet, private) that are devoid of factors that may lead to interruptions and a sense of urgency to complete encounters; and (3) emphasizing to those administering SBI the importance of delivering the complete protocol.

Additional issues should be considered when designing SBI delivery models that are alternatives to using medically trained providers as they have implications for the quality of both screening and BIs. First, while most health care providers would have access to the individual’s medical records and could integrate relevant information about medical conditions into the BI, the HEs lacked this information in a third of cases because they did not ask about it. Second, it is important when considering compensation structures for those delivering SBI that they prioritize the in-depth and insightful dialog in personalized BIs to encourage behavior change. Compensation should be structured so as not to disincentivize MI by prioritizing the volume of completed screenings relative to the comprehensive delivery of BIs. Third, both cultural factors associated with the population to be screened and the characteristics of the intervention should be carefully considered. If there is a significant mismatch between patients’ education level, language skills, and norms around discussing alcohol consumption, alternate screening instruments or methods may need to be used (e.g., ensuring the interviews are conducted in privacy). Ensuring that screening instruments are culturally appropriate to the population may help reduce problems with comprehension and administrators’ temptation to reword them.

### 4.1 Limitations

This study was conducted in conditions that may have created particular challenges for the HEs conducting the SBI. Both the HEs and pseudo-patients were typically masked, and interviews were conducted in often crowded public spaces that precluded privacy and confidentiality. HEs lacked access to patients’ medical records, which may have limited their ability to personalize their BIs. HEs had no control over when their interactions might be interrupted by patients’ appointments. In the absence of these contextual constraints, the pseudo-patients’ target scores may have been more accurately assessed by HEs and the BIs more complete. In addition to the contextual challenges associated with the intervention, there are limitations to the study methodology. The sample of SBI encounters is small, which limited our analyses. We were not able to fully examine whether characteristics of HEs, pseudo-patients, or different health facilities related to the quality and comprehensiveness of the SBI delivered. Replicating this SBI model with larger samples and under conditions that permit privacy and adequate time to administer BIs as recommended would inform these issues.

## 5 Conclusion

Using trained nonmedical persons to deliver SBI holds promise for increasing its reach, especially in LMICs where cost-effective methods for identifying and intervening with high-risk drinkers are urgently needed. However, future efforts to develop models to expand SBI need to overcome the obstacles identified in this study. This includes developing training for both screening and BI to reduce errors and improve quality of delivery. For screening, this means emphasizing that all screening items and response options should be administered exactly as they are written. When patients’ responses to screening questions are unclear, the response options should be repeated and, if necessary, further probes used to obtain an answer that can be coded accurately. No responses should be inferred from answers to previous questions. While providing general alcohol information is often the form of BIs for moderate drinkers, training needs to include adequate practice on the more complex process of eliciting personally relevant information from risky drinkers to explore their motivations and concerns around reducing their drinking and help them develop personalized goals and strategies. In planning where to locate an SBI program, careful attention should be paid to avoiding contexts that may negatively affect the SBI process. Locations that are crowded, noisy, lack spaces that afford privacy, and where interruptions are likely may make those being screened reluctant to disclose sensitive information about their drinking. They also impede the ability to have a dialog, and may put pressure on those administering the screening to conduct the SBI expeditiously. Relatedly, incentive structures for those administering SBI may also encourage hasty interactions. Thus, they need to be developed carefully to avoid incentivizing volume of SBIs conducted over quality. Finally, during the initial planning phase careful attention should be paid to the alignment between the education level, language skills, and norms of the population to be screened and the screening instruments to be used in SBI. This can reduce the chances that those administering SBI feel they need to reword items or make inferences that can affect the validity of the screening.

One approach that may address a number of these issues is the electronic delivery of SBI (eSBI) online, by smartphone app or through other automated platforms, that might afford high-risk drinkers a greater measure of privacy as well as reduce errors in the administration and scoring of screening measures. Research indicates a high degree of patient acceptability for eSBI and suggests that the strategy, especially when the screening is self-administered, is likely to reduce discomfort or embarrassment associated with answering questions about sensitive behaviors (33–35). Importantly, research also suggests that alcohol eSBI may be as effective as traditional SBI in reducing hazardous alcohol use, at least in the short-term (36–38), and produces similar effect sizes (13), although most of the pertinent studies have been conducted in high-income countries. Although electronic modes of delivery for SBI may address concerns around privacy, patient discomfort/embarrassment, and deviations in screening administration and scoring, it remains to be seen if BIs delivered electronically can generate the same degree of rapport and empathy and in-depth exploration of the factors related to successfully reducing drinking as those administered face to face, and whether they produce similar results in LMICs.

## Data Availability

The raw quantitative data (de-identified AUDIT data and SBI checklist data) supporting the conclusions of this article will be made available by the authors without undue reservation. Participants in the qualitative interviews were informed in the IRB-approved consent script that to protect their anonymity and confidentiality, the audio recordings and transcripts would be maintained on secure PIRE computer systems and only be accessible to project staff; thus, the authors cannot make these data available.

## Footnotes

^1^ https://www.scalaproject.eu/index.php
