# Supplementary material for: Investigating the implementation of a novel approach to alcohol screening and brief intervention in Mexico: a mixed-methods study using pseudo-patients
Source: Front Public Health. 2024 Oct 23;12:1416190. doi: 10.3389/fpubh.2024.1416190 (PMC11538022; doi:10.3389/fpubh.2024.1416190)
Supplement: Supplementary file 1 [file Data_Sheet_1.docx]

Supplementary Material

**Pseudo-Patient Interview Guide**

**Verbal consent script: To be provided in text to participant prior to interview and summarized at introduction.**

Thank you so much for agreeing to participate in this debriefing interview for your role in this innovative study.

Try to find a private, quiet space for our conversation. While we won’t be needing to ask any personal questions, we would like to have a safe and quiet space for a good recording.

You are being asked to participate in an interview to inform a study on the Escalemos project in Mexico. This interview is being led by the Pacific Institute for Research Evaluation (PIRE) and is funded by the AB InBev Foundation.  Your interview will be used to help PIRE understand how this project works from the perspective of the pseudo-patients. The purpose of the interview is not to evaluate your individual performance but to hear about your insights and experience.

Your participation consists of one recorded virtual interview of about an hour. You may decide to stop participating at any time.

Everything shared during this interview is confidential, that is, things you share with us won’t be reported with your name or anything that could identify you. Only PIRE study staff will have access to your full interview and its recording. However, keep in mind that the number of pseudo-patients is small and while we seek to assure that anything you say will not be identifiable in any resulting publications or reports, it is still possible that things you say could identify you to your colleagues or supervisors at TG Consultoría or elsewhere. For this reason, please take care not to report anything that you fear may bring you negative consequences. We can also turn off the recording feature at any time upon your request.

During the interviews, we will talk about some experiences with your collaboration in the project, your reflections about the strengths of Escalemos, and areas for improvement.  If time allows we’d also like to hear your reflections about the expansion of Escalemos into other platforms and locations.

You will receive a $20 digital gift card for participating.

Before we start, just remember:

- There are no right or wrong answers to these questions.
- This is NOT a performance evaluation of your work as a pseudo-patient. We want to understand what you think about this project, what you’ve thought and learned.

**May I start the recording?**

*Primary queries are numbered with possible probes in a.b.c form and parenthesis. Questions in BOLD are the core questions of inquiry for our study.*

1. **Just briefly, so we can get to know each other (again), tell me a bit about your background and how you became a pseudo-patient for our study?** (What other/related jobs do you have? Have you been a part of the Mystery Shopping project?)
   1. **What did you enjoy or not enjoy about this job as a pseudo-patient?** (What was hard or easy for you)?
   2. **What, if anything, did you learn from participating in this project?** (Was the alcohol information new? Anything about the Mexican healthcare system?)
2. **Let’s talk about the interactions you had as a pseudo-patient with the health educators.**
   1. **Can you tell me a story about a visit where the screening and brief intervention seemed to work out well?** (What made it a good or positive interaction? Why?) *(probe for environment, qualities of the educator, information provided (or not) by HE, etc.)*
      1. In that positive interaction, how did they get you to talk about changing your problem drinking? Did they even do that?
   2. **How about a story about a visit where the interaction did not go so well?** (What do you think contributed to that interaction being difficult? Why? *(probe for environment, qualities of the educator, information provided (or not) by HE etc.)*
   3. (if not addressed) In your experience, what were the barriers to a successful screening and brief intervention *(Probe for: noise, personal space/privacy, masks, etc.)?* In general, what do you think made interactions difficult or fail?
   4. (if not addressed) In general, what helped make the interactions work well when they were successful?
   5. **Thinking about what you observed as you waited to be approached by a health educator, how did others seem to be responding to the health educator?**
      1. Did you observe others having challenges interacting with the health educators? What challenges did they face?
      2. Did certain kinds of people seem to be approached more by the health educators? Did they participate more?
      3. Did people who were approached by the health educators seem receptive to participating in the interaction? How could you tell?
3. **A brief intervention is meant for people who score high on the AUDIT screening. Based on your experience as a pseudo-patient who scored high enough to receive a BI, what do you think are the key elements to this intervention?** *Probe: What should the HE be doing in that moment? How would you define it?*

**Remember there’s no right or wrong answers.** *(if mentioned one before, recall that one)*

- 1. Did any of the health educators provide very personalized advice and set personal goals with you? How did that happen?
  2. Did they ask about how ready you were to change and what concerns you might have about reducing consumption?
  3. Did they tailor suggestions for reducing alcohol consumption to your life specifically? What did that feel like? Describe it.
  4. **Our research team has learned that frequently, health educators are not providing brief interventions with people who score high for risky or excessive drinking and are not having conversations about how they can reduce their use in a way that’s personalized to the individual.**
     1. Why do you think this happened to you? What barriers got in the way of brief interventions?

1. **Now we’d like to hear your thoughts about how other Zacatecans or even Mexicans might respond to this program. Think especially about those you know who drink and drink more than is healthy.** *(Note this section may already be covered in previous discussion. If so, confirm their previous response and see if they’d like to add more)*
   1. **If you had been a real patient receiving the screening while waiting for a medical appointment, how do you think you would feel being asked those questions in those environments? (**Would it feel strange or odd? Is it common to be asked questions like that in a place where others can hear your response and the advice given? Is it common to talk about alcohol use in a healthcare setting?)
      1. **How do you feel about using a healthcare setting and health educators looking like professional medical staff?** Do you think others would find it easier answering those questions or feel confused?
   2. **Thinking about typical Zacatecan patients– especially a typical drinker -- were there questions that you think people would find hard to answer**? What were they and why? (What questions, if any, were hard to understand? Why? Who might find them hard to understand or answer?)
   3. **Most participants receive some advice about “responsible drinking”. What do you think about the responsible drinking advice you received?** (drinking water between drinks, etc.) How relevant is this information to people like you or those you know, especially those who may drink more than is healthy? Why or why not?
   4. **Again, thinking about those you know who drink like you were asked to report as a pseudo-patient – that is, as someone who scores as a risky/excessive drinker. How do you think these individuals would respond to the brief intervention portion of the interaction?** **(**Where it was recommended that they cut back, helping to find ways in a personalized fashion to cut back, trying to identify your readiness to change, etc.)
   5. Do you think they’d be honest about their willingness to reduce consumption? Why? Do you think they’d apply the advice they receive? Why?
   6. **From the perspective of someone you know who may drink more than they should, do you think that participating in Escalemos would help them change? Why? Why not?**
      1. Do you think the people who most need the intervention are getting it? Why or why not?
      2. How can more people who need to cut back their drinking be reached?
      3. What can help people get access to intervention or treatment when they need it?
      4. How do you think people can ask for help with cutting down their harmful drinking in Zacatecas? Can medical providers offer that help? Why or why not?

*(Only ask the next question if there is time and the participant appears engaged.)*

1. Some are interested in moving Escalemos to online implementation. Based upon your experience as a pseudo-patient and observing others being screened, as well as your experience as a Mexican and Zacatecan, what do you think the strengths and weakness are for this type of implementation?
   1. What do you think would be the benefits of doing this online?
      1. For example, what if you gave yourself the screening online at home, or before a medical encounter? Would people be more or less likely to answer the questions accurately? Do you think people prefer having someone in person asking those questions? Would it feel safer? Would you trust the information more from a person who you interact with or an app or system that provides feedback electronically? If you would prefer a live person, would it matter to you if the interaction occurred in person or remotely such as in a video or telephone call?
   2. What would make Escalemos implementation hard if it was exclusively online?
      1. Might comprehension of the questions be an issue for some people?

1. Are there any other comments that you would like to share that might help us understand your experience with this project?
